# Case 6/2017 - Extensive Giant Left Coronary Artery Aneurysm Due to Kawasaki Vasculitis in Asymptomatic 48-Year-Old Man

**DOI:** 10.5935/abc.20170157

**Published:** 2017-11

**Authors:** Edmar Atik, Roberto Kalil Filho, Fabio Jatene, Júlio Cesar S Marino

**Affiliations:** Hospital Sírio Libanês de São Paulo, São Paulo, SP - Brazil

**Keywords:** Left Coronary Artery Aneurysm, Kawasaky Disease, Coronary Artery Disease

**Clinical data:** Three months ago, there were retrosternal pain, fatigue and tachycardia (170 bpm) for atrial flutter reversed with amiodarone. Thoracic tomography revealed a giant aneurysm of the left coronary artery. Corrected atrial septal defect at 3 years of age. He has active life with moderate sport. Corrected cryptorchidism at 12 years of age with impairment of fertility.

Physical examination: good general condition, eupneic, acyanotic, normal pulses on the 4 limbs. Weight: 88 Kgs, H: 172 cm, *upper extremity blood pressure*: 130/80 mmHg, HR: 60 bpm. Aorta not palpated at the suprasternal notch.

Precordium: non-palpable ictus cordis, without systolic impulses. Hypofonetic heart sounds, without heart murmurs. Unpalpable liver and clean lungs.

## Additional Examinations

**Electrocardiogram:** sinus rhythm, without cavitary overloads, complete right bundle branch block and 1st degree atrioventricular block. PR: 0.22, QRS: 0.109 with complexes rSr´ in V1 and RS in V6; AP = + 0º, AQRS = + 220º, AT = + 66º.

**Figure 1:**
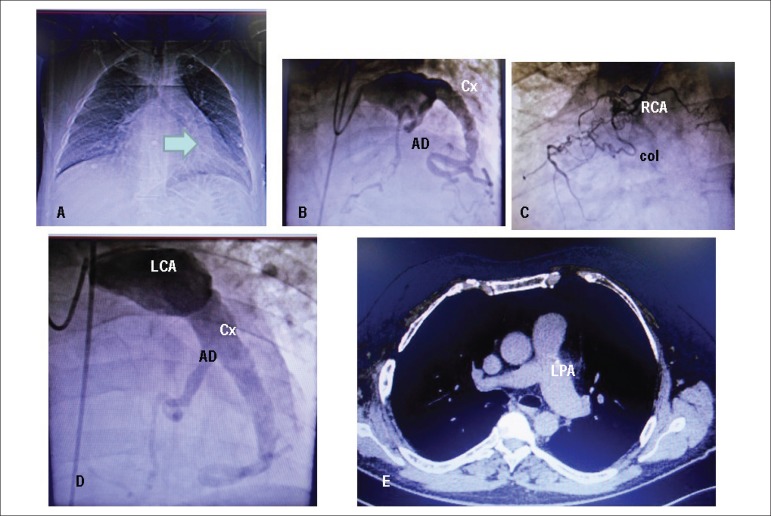
Chest X-ray in PA highlights normal cardiac area and pulmonary vascular tissue. Left ventricular border shows a dense rectilinear image that corresponds to the left coronary artery aneurysm (arrow). Coronary angiography points out the aneurysm of the trunk of the left coronary artery and the circumflex artery (B and D), the obstruction of the anterior descending artery (B and D) and the right coronary artery (C). It is observed filling of this artery, with total obstruction, from the left coronary and the distal part of the AD. Chest tomography shows dilatation of the left pulmonary artery, and normal caliber of ascending and descending aorta (E).

**Chest X-ray:** normal cardiac area (cardiothoracic index = 0.50) and linear vascular image with increased density bordering the ventricular arch (Figure 1A).

**Echocardiogram:** normal cardiac chambers except for discrete left atrial enlargement, normal biventricular function. Dilatation of the left coronary artery corresponding to the circumflex artery at the atrioventricular junction in the anterolateral wall of the left ventricle, measuring 40 mm. Aorta = 34 mm, LA = 46, RV = 25, LV = 47, septum = posterior wall = 10 mm, LVEF = 68%.

**Holter:** Sinus rhythm, heart rate = 56 to 100, mean = 72 bpm. Polymorphic ventricular extrasystoles, bigemy, frequent, especially at dawn and morning. 1st-degree atrioventricular block, PR = 0.26, alternating with normal AV conduction. Absence of changes in ventricular repolarization and symptoms.

**Coronary angiography:** right coronary occluded at the origin, with intracoronary collateral circulation. Trunk of the left coronary artery with large aneurysmal dilatation and parietal irregularities with whirling flow. Anterior descending artery exhibits occlusion at the origin and distal opacification by ipsilateral collaterals. Circumflex artery exhibits large ectasia and parietal irregularities. It emits ipsilateral collateral circulation to anterior descending and right coronary. Left ventriculography exhibits preserved diastolic volume and discrete anteromedial hypokinesia, with competent mitral valve (Figure 1B, C, D).

**Computed tomography of the thorax** shows a large elongated sacculation of vascular origin in the subaortic region, next to the topography of the circumflex artery, 10.0 cm on the largest axis (Figure 1E).

**Clinical Diagnosis:** Extensive giant aneurysm of the left coronary artery from the trunk to the middle third of the circumflex with total obstruction of the anterior descending and right coronary arteries.

**Clinical Reasoning:** In asymptomatic patient with previous correction of simple cardiac defect (ASD at 3 years of age), recent clinical features of atrial flutter and complete right bundle branch block did not imply the existence of coronary pathology with exaggerated dilation of the coronary arteries without ischemia and/or ventricular dysfunction. This diagnosis was established by imaging tests, particularly by chest angiotomography and coronary angiography. The chest radiograph, if better analyzed, could have opened the diagnostic suspicion and the echocardiogram emphasized the diagnosis.

**Differential diagnosis:** the presumptive cause of the unusual aneurysm of the coronary arteries, due to its extension and magnitude, was oriented to a previous arteritis process. Given the concomitant presence of clear obstructions, especially of the right coronary artery, the immediate assumption was that of Kawasaki syndrome, which occurs in the infantile age and progresses to significant alterations of risk still in the child as myocardial infarction, rupture of aneurisms and sudden death. The evolution in adulthood is rare, but possible because the aneurysm, even sharp, can evolve silently without causing harm, as noted. Other causes of arteritis refer to Takayasu syndrome, connective tissue diseases (polyarteritis nodosa, lupus and scleroderma), atherosclerosis, and infections such as syphilis.

**Conduct:** There was a preventive surgical indication due to the magnitude of the coronary alterations. The aneurysm of the left coronary trunk was 45 mm in diameter with organized thrombus in the interior. It was done thrombectomy and interposition of 10 mm dacron tube in its proximal and distal stumps. Bypass from the aorta to the anterior descending artery, extracted from the left thigh (rare site of whole vein, since the others presented inflammatory tissue without perviability, including the left mammary artery). Prolonged surgery (5:40 pm ECC and 151-minute ischemia) with the exclusion of the left marginal that emerged from the large aneurysm, caused cardiogenic shock with ECMO continuity for 11 days, 22-day intra-aortic balloon and impaired ventricular function, but with progressive improvement of 34 to 58%, without enlargement of cardiac cavities, but inferior and lateral akinesia. Histological study of the coronary artery fragment revealed thick arterial wall with fibrosis, calcification, epithelioid granulomas with giant multinucleated cells, characteristics of Kawasaki vasculitis.

**Comments:** Anatomical normality after correction of coronary aneurysms brought relief despite a myocardial ischemic process due to the interruption of emergent vessels. In the literature, 28 cases of coronary aneurysms in adults were reported in a period of 49 years,^1^ and most of them (68%) were operated by aneurysm ligation and coronary artery bypass grafts, with good progression in the majority (95%). There have been reports of percutaneous intervention in localized aneurysms^2^ of less than 10 mm, as well as cases in clinical treatment with anticoagulants, but with an unfavorable outcome (62.5%).
